# EZH2 Inhibition Interferes With the Activation of Type I Interferon Signaling Pathway and Ameliorates Lupus Nephritis in NZB/NZW F1 Mice

**DOI:** 10.3389/fimmu.2021.653989

**Published:** 2021-03-26

**Authors:** Lingling Wu, Xiaoyue Jiang, Chaojun Qi, Chunyan Zhang, Bo Qu, Nan Shen

**Affiliations:** ^1^ Department of Rheumatology, Ren Ji Hospital, School of Medicine, Shanghai Jiao Tong University, Shanghai, China; ^2^ Department of Nephrology, Molecular Cell Lab for Kidney Disease, Ren Ji Hospital, School of Medicine, Shanghai Jiao Tong University, Shanghai, China; ^3^ Shenzhen Futian Hospital for Rheumatic Diseases, Shenzhen, China; ^4^ State Key Laboratory of Oncogenes and Related Genes, Shanghai Cancer Institute, Ren Ji Hospital, School of Medicine, Shanghai Jiao Tong University, Shanghai, China; ^5^ Center for Autoimmune Genomics and Etiology (CAGE), Cincinnati Children’s Hospital Medical Center, Cincinnati, OH, United States; ^6^ Department of Pediatrics, University of Cincinnati College of Medicine, Cincinnati, OH, United States

**Keywords:** systemic lupus erythematosus, enhancer of zeste homolog 2, type I interferon, epigenetics, multivalent therapeutic target

## Abstract

Enhancer of zeste homolog 2 (EZH2) is a histone-lysine N-methyltransferase mediating trimethylation of H3K27, which represses gene expression and is critical to immune regulation. Inhibition of EZH2 is proved to have the potential of treating many diseases. However, whether inhibition of EZH2 affects type I interferon (IFN-I) signaling pathway, the abnormality of which is an important pathogenic mechanism for SLE, is still elusive. Here, we report, unexpectedly, a positive regulatory function of EZH2 in IFN-I signaling pathway, which contributes to the overactivation of IFN-I signaling pathway in SLE. We show that the expression of EZH2 was upregulated and positively correlated with the overexpression of interferon stimulated genes (ISGs) in both peripheral blood mononuclear cells and renal tissues of SLE patients. *In vitro* inhibition of EZH2 by either siRNAs or chemical inhibitors reduced the phosphorylation of STAT1 and the induction of ISGs stimulated by IFN-I. Additionally, inhibition of EZH2 interfered with the *in vivo* and *ex vivo* activation of IFN-I signaling pathway elicited by intravenous injection of adenovirus vector expressing mouse IFN-α5 and exogeneous stimulation with IFN-α, respectively. We evaluated the therapeutic effects of EZH2 inhibitor in NZB/NZW F1 mice which depend on IFN-I signaling pathway for the lupus-like disease development. Administration of EZH2 inhibitor prolonged the survival, reduced the levels of anti-dsDNA autoantibodies, and improved lupus nephritis of the mice. What’s more, EZH2 inhibitor attenuated the expression of ISGs in the kidneys of these mice. In summary, we show that excessive EZH2 contributes to the overactivation of IFN-I signaling pathway in SLE. EZH2 inhibitor has the potential to inhibit IFN-I signaling pathway and alleviate lupus nephritis. Additionally, diverse disease driving pathways exist among systemic lupus erythematosus (SLE) patient, and even in the same patients. Common regulators of different pathogenic pathways can be multivalent therapeutic targets. Together with previous studies showing EZH2 is involved in T-cell and B-cell mediated immune responses, EZH2 could be a potent multivalent therapeutic target for SLE.

## Introduction

Systemic lupus erythematosus (SLE) is a systemic autoimmune disease with damage to multiple organs (1). Although amazing progress has been made in treating SLE, the development of new SLE therapies is slow and inadequate, since belimumab is the only drug approved by FDA during the past ~50 years, and corticosteroids together with immunosuppressive agents are still the first-line medicine for SLE (2, 3). One of the obstacles result from the heterogeneity of SLE pathogenesis, suggesting the potential of individualized treatments targeting specific pathogenic pathways (1, 4). In contrast, common regulators of different SLE pathogenic pathways can be multivalent therapeutic targets (1). Drugs targeting common regulators can benefit more patients, the development of which requests deep understanding of the regulation of critical SLE pathogenic pathways.

Epigenetics, through the combination of DNA methylation, histone modifications, and non-coding RNAs, controls gene expression in a reversible way, without altering the genome (4). Dysregulated epigenetic modifications is involved in SLE pathogenesis (5, 6). Widespread and severe DNA hypomethylation near genes associated with type I interferon (IFN-I) signaling pathway—a critical SLE pathogenic pathway—in the immune cells from SLE patients contributes to the overactivation of IFN-I signaling pathway (7–9). Epigenetic changes are responsible for abnormal T cell activation—another critical SLE pathogenic pathway—in SLE patients (10). Additionally, IL2 silencing in T cells from SLE patients, a well-recognized defect of SLE T cells, depends on histone deacetylation and DNA hypermethylation (11). Therefore, considering that epigenetic modifications are reversible, epigenetic modification agents constitute novel drug candidates for SLE. Indeed, some anti-rheumatic drugs, such as methotrexate and mycophenolate mofetil, have epigenetic modification activities (12, 13). Additionally, histone modification enzymes can mediate post-translational modifications of non-histone proteins to regulate cellular signal transduction, which extends the mode of action and the potential of epigenetic therapies (14).

Enhancer of zeste homolog 2 (EZH2) is a histone-lysine N-methyltransferase mediating di- and trimethylation of H3K27, which represses gene expression (15). Abnormal functions of EZH2 are associated with many types of cancers, and EZH2 inhibitors serve as promising drug candidates (15). As to SLE, excessive expression of EZH2 results in epigenetic landscape shift in naïve CD4^+^ T cells, which favors proinflammatory responses, while opposes inhibitory functions (10); EZH2 promotes the hypomethylation of genes involved in leukocyte adhesion and migration in CD4^+^ T cells (16); EZH2 mediated methionine’s immune regulatory function in facilitating the differentiation of B cells into plasma blasts in SLE (17); inhibiting EZH2 reduced lymphoproliferation, the number of double-negative (DN) T cells, and ameliorated lupus-like disease in MRL/lpr mice, in which the disease is driven by the defects of cell death and the overactivation of adaptive immune responses (18, 19). Besides its histone modification activity, EZH2 can also regulate proinflammatory signaling pathways through direct methylation of essential transcription factors, such as p53, NF-κB, and STAT3 (20). However, we don’t know if EZH2 participates in IFN-I signaling pathway, which is overactivated in more than 60% of SLE patients and critical to the development of SLE (21, 22).

Here, we investigated the role of EZH2 in IFN-I signaling pathway and the therapeutic effects of EZH2 inhibitor on lupus nephritis in NZB/NZW F1 mice, a model depends on IFN-I, which differs from MRL/lpr mice (19, 23). Since the blockade of IFN-I signaling has shown positive therapeutic effects for SLE (24), the demonstration of inhibiting EZH2 could interfere with IFN-I signaling, together with the previous findings that inhibiting EZH2 could reverse SLE T-cell abnormalities, suggests that EZH2 is a potent multivalent therapeutic target which may benefit broad SLE patients.

## Materials and methods

### Human Samples

PBMCs from 30 SLE patients and 30 healthy controls were from Ren Ji Hospital Biobank, Shanghai Jiao Tong University School of Medicine. The study was approved by the Research Ethics Board of Ren Ji Hospital. Written Informed consent was obtained from all subjects. All SLE patients met the American College of Rheumatology (ACR) 1997 revised criteria or 2012 SLICC criteria for SLE (25, 26). The clinical information of the study subjects is in **Table S1**.

### Mice

C57BL/6N mice were purchased from Vital River Laboratories. NZB/BINJ and NZW/LacJ mice were purchased from The Jackson Laboratory. All animals were maintained in a specific pathogen free facility at Ren Ji Hospital under a 12-h light/12-h dark cycle with free access to water and standard mouse diet. NZB/NZW F1 mice were a hybrid cross between NZB/BINJ female and NZW/LacJ male. Mouse studies were approved by the Animal Care Committee of Ren Ji Hospital. All the animal experiments were performed according to the protocols approved by the Institutional Animal Care and Use Committee at Ren Ji Hospital, Shanghai Jiao Tong University School of Medicine.

For *ex vivo* or *in vivo* assessment of the effect of GSK126 on IFN-I signaling, 8 weeks old female C57BL/6N mice were used. The *in vivo* activation of IFN-I signaling was achieved by intravenously injecting the mice with 10^9^ pfu of IFNα5-encoding adenovirus particles (ViGene Biosciences). GSK126 (50 mg/kg) (Selleck Chemicals) was administrated intraperitoneally. For *ex vivo* stimulation of splenocytes, 1,000 U/ml of universal type I IFN (universal IFN-I) (R&D System) was used.

For evaluation of the therapeutic effects of GSK126, 24–28 weeks old female NZB/NZW F1 mice were randomized and grouped based on their urinary protein levels. The mice were treated intraperitoneally with GSK126 (50 mg/kg) or vehicles every other day for 4 weeks. Peripheral blood was collected with tubes containing ethylenediaminetetraacetic acid and plasma was isolated. Anti-dsDNA autoantibody was measured with Mouse anti-dsDNA IgG-specific ELISA Kit (Alpha Diagnostic International). When needed, the mice were kept in separated metabolic cages and 24-h urine was collected. Urinary protein was measured with BCA Protein Assay kit (TIANGEN). Kidneys were harvested 2 days after the last treatment and fixed in 4% formaldehyde solution. Then they were embedded in paraffin. Five µm thick sections were taken and stained with hematoxylin and eosin (H&E). Blinded assessment of renal pathological scores was blindly performed by a senior renal pathologist, as previously described (27). Briefly, we screen the glomeruli for hypertrophy, proliferative changes, crescent formation, hyaline deposits, fibrosis/sclerosis, and basement membrane thickening. Glomerulonephritis severity was graded on a 0–4 scale as described in (27). Tubulointerstitial nephritis severity was also graded on a 0–4 scale considering the extent of tubular atrophy, inflammatory infiltrates, and interstitial fibrosis, as detailed in (27). The precipitation of immune complex was evaluated by standard immunofluorescence with anti-mouse IgG2a (Santa Cruz Biotechnology).

### Cell Culture

THP-1 cells (Cell Bank, Shanghai Institutes for Biological Sciences, Chinese Academy of Sciences) and monocytes were maintained in RPMI 1640 medium supplemented with 10% (v/v) fetal bovine serum (Gibco), 2 mM L-Glutamine, 1 mM sodium pyruvate, and 10 mM HEPES at 37°C in a humidified atmosphere containing 5% CO2.

PBMCs were isolated using Ficoll-Paque PLUS (Cytiva). Monocytes (>98% purity) were sorted from the PBMCs with a BD FACSAriaTM III cell sorter (BD Biosciences). Monocytes were identified by anti-CD14 antibody (BD Biosciences).

For transient transfection, THP-1 cells (2 × 10^5^/well) were seeded in 24-well plate 24 h before transfection. SiRNAs (combination of four different EZH2 targeting siRNAs, 200 nM) were transfected with LipofectamineTM RNAiMAX (Invitrogen). Twenty-four hours later, the cells were stimulated with 1,000 U/ml of universal IFN-I, then collected for further analysis as shown in the figures. All siRNA sequences (GenePharma) are in **Table S2**.

For EZH2 inhibitor treatment, THP-1 cells were pretreated with 5 uM GSK126, 5 uM Dznep (Selleck Chemicals), or DMSO for 30 min, then 1,000 U/ml of universal IFN-I was added. Then, the cells were collected for further analysis as shown in the figures.

### Quantification of Gene Expression

Total RNA was extracted using TRIzolTM reagent (Invitrogen) and used to synthesize cDNA with PrimeScript RT Reagent kit (Takara Bio). Gene expression was quantified with SYBR Premix Ex Taq kit (Takara Bio). The amplification was performed on a QuantStudio 7 Flex Real-Time PCR System (Applied Biosystems). RPL13a was used as an internal control. The expression levels were calculated using 2^−△△Ct^ method. All primers (Sangon Biotech) are listed in **Table S3**. RNA-seq analysis is described in **Supplementary Methods**.

### Western Blot

Proteins were extracted with RIPA Lysis Buffer supplemented with Halt™ Protease and Phosphatase Inhibitor Cocktail (Thermo Fisher Scientific). The proteins (10 μg/sample) were separated by electrophoresis on 10% SDS PAGE followed by transferring to PVDF membranes (Millipore). Specific proteins were detected by the corresponding primary antibodies and secondary peroxidase-conjugated antibodies (**Table S4**). Bands were detected by Pierce ECL Western Blotting Substrate (Thermo Fisher Scientific). Images were taken by Tanon 5500 Imaging system. Densitometry quantification was by ImageJ software (NIH).

### Statistical Analysis

Data were analyzed and graphs were generated with GraphPad Prism (version 6.02). Non-parametric Mann-Whitney U-test, Spearman’s correlation test, Student’s paired t-test, two-way ANOVA, and log-rank test were applied as indicated. The difference was considered statistically significant when the P value was <0.05.

## Results

### EZH2 Overexpression Associates With the Overactivation of IFN-I Signaling Pathway in SLE

**Figure 1:**
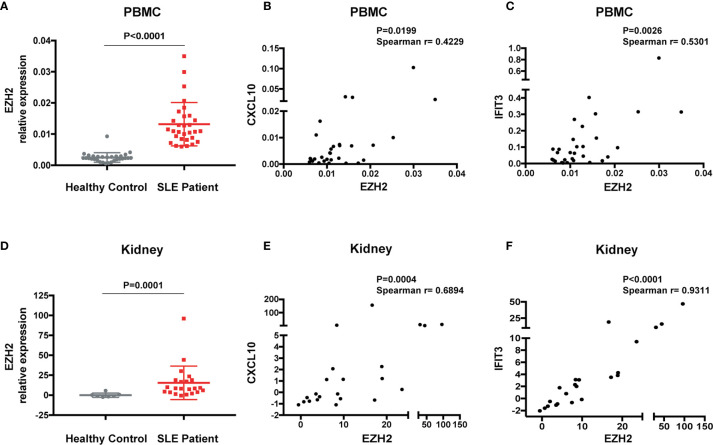
Overexpression of EZH2 associates with the upregulation of ISGs in SLE. **(A)** The levels of EZH2 mRNA in the PBMCs from SLE patients (n = 30) and healthy controls (n = 30). **(B, C)** Positive correlation between the expression levels of EZH2 and CXCL10 **(B)** and IFIT3 **(C)** in the PBMCs from SLE patients (n = 30). **(D–F)** RNA-seq analysis was previously performed using kidney biopsies from SLE patients (n = 22) and the carcinoma–adjacent normal kidney tissues (n = 7) (29). **(D)** The levels of EZH2 transcript in the kidney biopsies from SLE patients and in the carcinoma–adjacent normal kidney tissues. **(E, F)** Positive correlation between the expression levels of EZH2 and CXCL10 **(E)** and IFIT3 **(F)** in the kidney tissues from SLE patients. **(A, D)** data are presented as mean ± SEM. *P* values were determined by Mann-Whitney U-test. **(B, C, E, F)** Each dot represents individual patients. *P* values were determined by Spearman’s correlation test.

To explore the interaction between EZH2 and IFN-I signaling in SLE, we measured the expression of EZH2 and two representative disease-associated ISGs (CXCL10 and IFIT3) (28). EZH2 was expressed higher in the PBMCs from SLE patients (n = 30) than healthy controls (n = 30) (**Figure 1A**). The mRNA levels of EZH2 positively correlated with CXCL10 or IFIT3 (**Figures 1B, C**), respectively. Overexpression of ISGs is also present in renal tissues of lupus nephritis patients (30). Thus, we examined the expression of EZH2 and CXCL10, or IFIT3, in our renal tissue RNA-seq dataset (29). We found EZH2 was upregulated in renal biopsies from lupus nephritis patients as compared with normal para-carcinoma kidney tissues (**Figure 1D**); and there were significant positive correlations between the expression of EZH2 and CXCL10 or IFIT3 (**Figures 1E, F**), respectively. Thus, EZH2 overexpression associates with the overactivation of IFN-I signaling pathway in SLE.

### EZH2 Facilitates the Activation of IFN-I Signaling Pathway

In PBMCs from SLE patients, monocytes overexpress ISGs more than three times as much as T cells or B cells (31). By re-analyzing the published microarray data (32), we found EZH2 was significantly upregulated in monocytes from SLE patients (n = 58) as compared with healthy controls (n = 68) (**Figure S1A**) and positive correlations were found between the expression of EZH2 and CXCL10 or IFIT3 (**Figures S1B, C**), respectively. Therefore, we used monocytes for the following studies.

**Figure 2:**
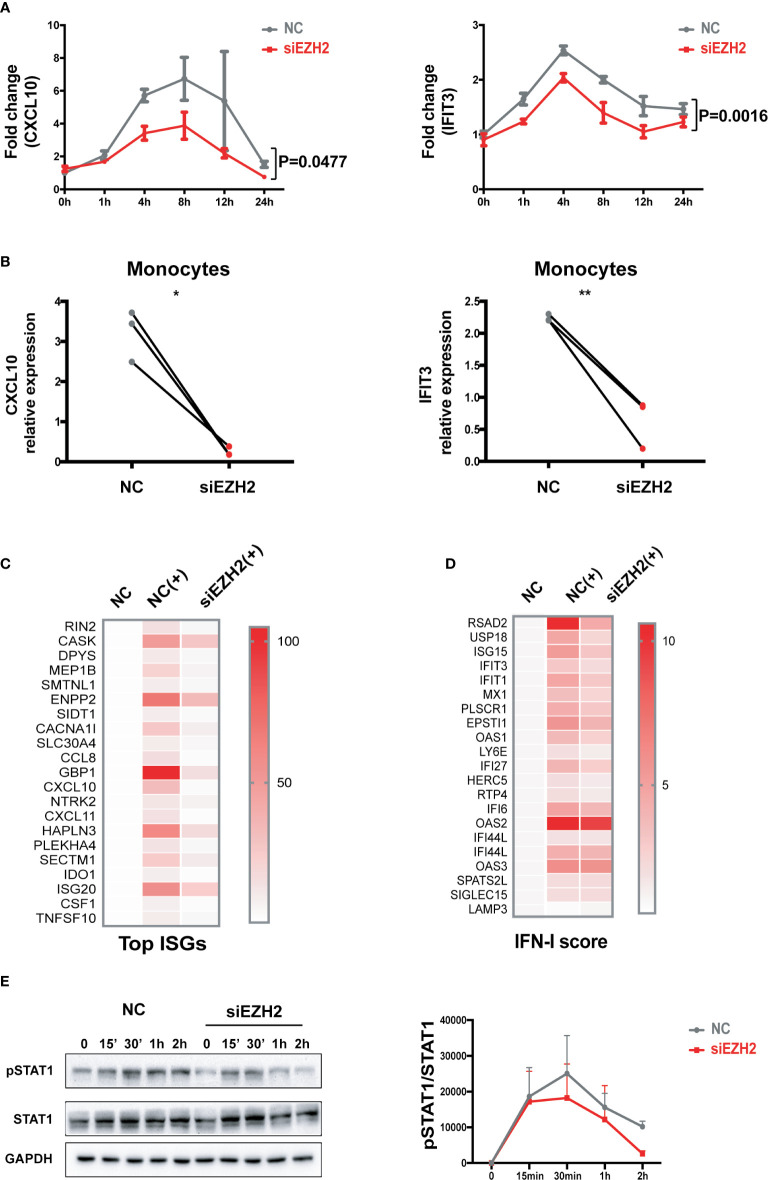
EZH2 regulates the activation of IFN-I signaling pathway. **(A)** THP-1 cells were transfected with 200 nM of EZH2 targeting siRNAs (siEZH2) or negative control siRNAs (NC). Twenty-four hours after transfection, THP-1 cells were stimulated with 1,000 U/ml of universal type I IFN. **(A)** The levels of CXCL10 mRNA and IFIT3 mRNA were measured at the indicate time points. **(B)** Primary monocytes were electroporated with 200 nM of EZH2 targeting siRNAs (siEZH2) or negative control siRNAs (NC). Twenty-four hours after electroporation, the cells were stimulated with 1,000 U/ml of universal type I IFN for 6 h. **(B)** The levels of CXCL10 mRNA and IFIT3 mRNA were measured. **(C, D)** RNA-seq analysis of the THP-1 cells stimulated with 1,000 U/ml of universal type I IFN with the perturbation of EZH2 or not. **(C)** Top 20 most affected ISGs. **(D)** The 21 ISGs constitute IFN signature scoring panel (33, 34). **(E)** IFN-I induced phosphorylation of STAT1 was determined in THP-1 cells transfected with 200 nM of siEZH2 or NC by Western bolt. Representative pictures of at least three independent experiments are shown. Band densities quantified by Image J **(A)** data are presented as mean ± SEM from at least three independent experiments. *P* values were determined by two-way ANOVA. **(B)** Each pair of dots represents an individual donor (n = 3). *P* values were determined by Student’s paired t-test. *P < 0.05, **P < 0.01. **(C, D)** Each row represents individual genes. Each column is an individual treatment, NC: negative control siRNAs without IFN-I stimulation; NC (+): negative control siRNAs with IFN-I stimulation; siEZH2 (+): EZH2 targeting siRNAs with IFN-I stimulation.

Silencing EZH2 inhibited the induction of CXCL10 and IFIT3 by IFN-I both in THP-1 cells (**Figures 2A** and **S2A**) and in primary monocytes (**Figures 2B** and **S2B**). We performed RNA-seq analysis of IFN-I stimulated THP-1 cells with or without the perturbation of EZH2 expression. Out of the 171 transcripts that were induced by IFN-I (fold change >2) in control group, 123 transcripts (71.9%) were significantly affected by EZH2 perturbation. Top 20 most affected transcripts are shown in **Figure 2C** (Reduction fold is listed in **Table S6**). A 21-gene IFN signature has been developed as a biomarker to evaluate the effectiveness of IFN-I blockade (35, 36). We found that 18 out of the 21 ISGs were affected by EZH2 perturbation (**Figure 2D**, reduction fold is listed in **Table S7**). Consistently, silencing EZH2 dampened IFN-I induced phosphorylation of STAT1, a key transcription factor in IFN-I signaling pathway (**Figure 2E**). These results indicate EZH2 facilitates the activation of IFN-I signaling pathway and excessive EZH2 contributes to the overactivation of IFN-I signaling pathway in SLE.

### GSK126 Inhibits IFN-I Signaling Pathway Both *in Vitro* and *in Vivo*

**Figure 3:**
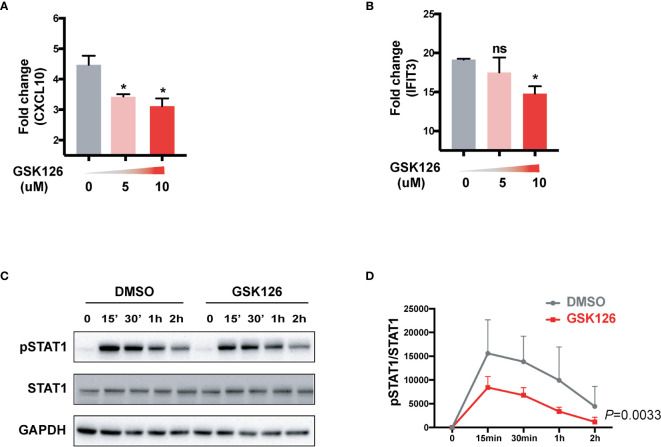
GSK126 attenuates the activation of IFN-I signaling pathway. **(A, B)** THP-1 cells were pretreated with different concentrations of GSK126 for 30 min, then stimulated with universal type I IFN (1,000 U/ml) for 4 h. The levels of CXCL10 mRNA **(A)** and IFIT3 mRNA **(B)** were measured. **(C, D)** IFN-I induced phosphorylation of STAT1 was determined in THP-1 cells pretreated with 5 μM of GSK126 by Western bolt. **(C)** Representative pictures of at least three independent experiments. **(D)** Band densities quantified by Image J **(A, B, D)** Data are presented as mean ± SEM. **(A, B)** *P* values were determined by Mann-Whitney U-test. *P < 0.05, ns, not significant. **(D)** *P* value was determined by 2-way ANOVA.

**Figure 4:**
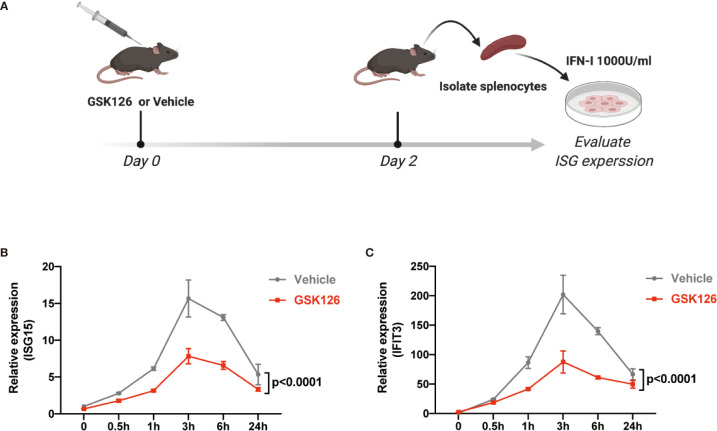
GSK126 inhibits *ex vivo* activation of IFN-I signaling pathway. **(A)** Experimental setting for the *ex vivo* tests. The mice were treated with a single dose of GSK126 (50 mg/kg, i.p.) and then sacrificed after 2 days. Splenocytes were isolated and stimulated with universal type I IFN (1,000 U/ml) for indicated time. **(B, C)** The levels of ISG15 mRNA **(B)** and IFIT3 mRNA **(C)** were measured. **(A)** Created with BioRender.com. **(B, C)** Data of three independent experiments are plotted and presented as mean ± SEM. *P* values were determined by two-way ANOVA.

**Figure 5:**
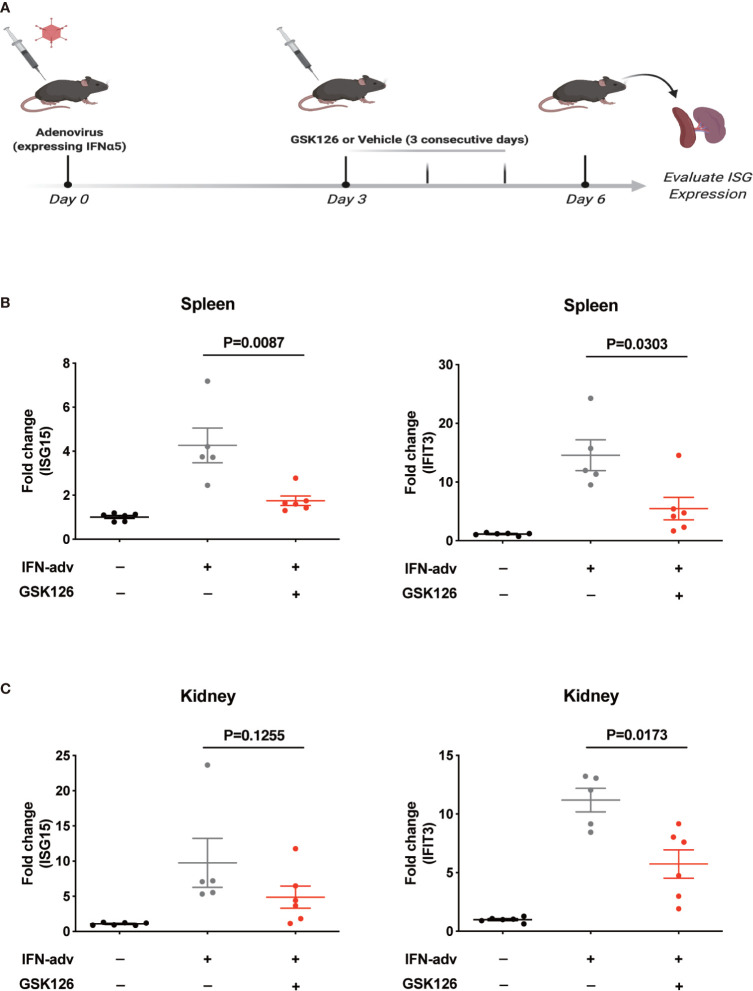
GSK126 inhibits *in vivo* activation of IFN-I signaling pathway. **(A)** Experimental setting for the *in vivo* tests. Each mouse received 10^9^ pfu of IFNα5-encoding adenovirus particles, or PBS (as control), for *in vivo* activation of IFN-I signaling pathways. Three days later, the mice received adenoviral particles were given GSK126 (50 mg/kg, i.p.), or vehicle, for 3 consecutive days. **(B)** The levels of ISG15 mRNA and IFIT3 mRNA in the spleens were measured. **(C)** The levels of ISG15 mRNA and IFIT3 mRNA in the kidneys were measured. **(A)** Created with BioRender.com. **(B, C)** Each dot represents individual mice (n ≥ 5 for each group). *P* values were determined by Mann-Whitney U-test.

We found GSK126, a highly specific competitive inhibitor of EZH2 (33), exerted an inhibitory effect on the activation of IFN-I signaling pathway (**Figures 3A, B**), which resembled the effects of EZH2 siRNAs. Similar results were obtained using another EZH2 inhibitor, DZNep (**Figures S3A, B**) (34). Both GSK126 (**Figures 3C, D**) and DZNep (**Figures S3C, D**) inhibited IFN-I induced phosphorylation of STAT1. Additionally, we isolated the splenocytes from the mice pretreated with GSK126 for 2 days, stimulated the cells with IFN-I, and measured the induction of ISGs at different time points (**Figure 4A**). The induction of ISGs was dramatically reduced by GSK126 as compared with vehicle (**Figures 4B, C**). To prove the *in vivo* activity of GSK126, we treated the mice that received IFN-α5 expressing adenoviral particles with GSK126 or vehicle for 3 consecutive days (**Figure 5A**). We found the induction of ISGs in spleens and kidneys was reduced by GSK126 as compared with vehicle (**Figures 5B, C**). Thus, EZH2 inhibitor, here represented by GSK126, could be used to inhibit the *in vivo* activation of IFN-I signaling pathway, indicating its potential application in SLE.

### GSK126 Alleviates Lupus Nephritis in NZB/NZW F1 Mice

**Figure 6:**
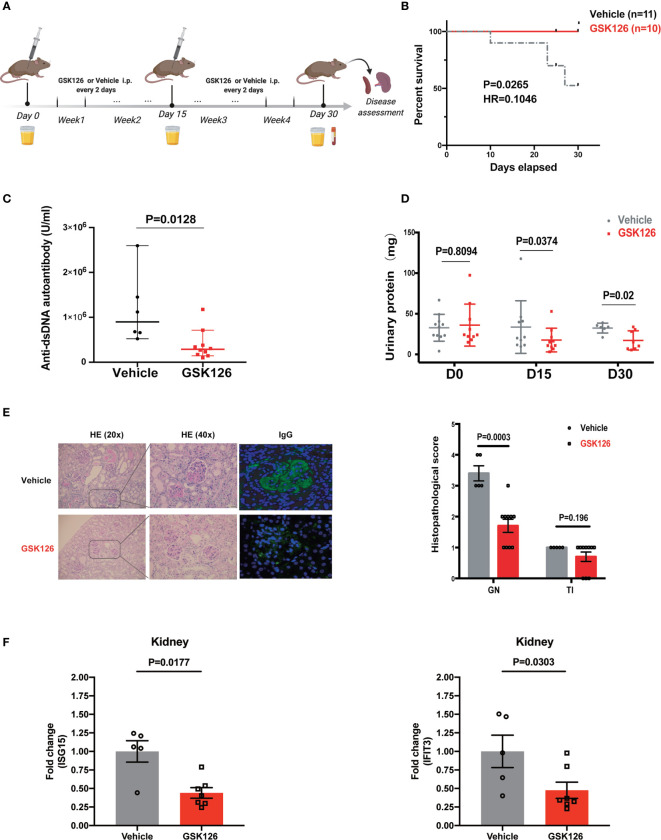
GSK126 alleviates lupus nephritis in NZW/NZB F1 mice. **(A)** Experimental setting. Briefly, 24–28 weeks old female NZB/NZW F1 mice were randomized and grouped based on their urinary protein levels. The mice were treated with GSK126 (50 mg/kg, i.p.), or vehicles, every other day for 4 weeks. Peripheral blood and 24-h urine were collected at indicated time points. Kidneys were harvested 2 days after the last treatment for histopathological analysis. **(B)** Kaplan-Meier survival curve (n ≥ 10 for each group). **(C)** Anti-dsDNA autoantibody in the plasma. **(D)** Twenty-four-hour urinary protein. **(E)** Representative pictures of renal H&E staining and immunofluorescent staining of glomerular deposition of immunocomplex. Renal histopathological scores were evaluated for the mice in GSK126 group (n = 10) and vehicle group (n = 5). GN, glomerulonephritis; TI, tubulointerstitial nephritis. **(F)** The levels of ISG15 mRNA and IFIT3 mRNA in the kidneys from the mice treated with GSK126 or vehicle. **(A)** Created with BioRender.com. **(B)** *P* value was determined by Log-rank test. (**C–E** right panel, **F**) Each dot represents individual mice. Data are presented as mean ± SEM. *P* values were determined by Mann-Whitney U-test.

We explored if EZH2 inhibitor could treat IFN-I-driven organ damage (lupus nephritis) in SLE mouse model. We explored the therapeutic effects of GSK126 on lupus nephritis in NZB/NZW F1 mice (**Figure 6A**), in which IFN-I is critical to the development of SLE and lupus nephritis (37–39). GSK126 significantly improved the survival of NZB/NZW F1 mice, although administrated in late stage of disease (**Figure 6B**). GSK126 reduced the titers of anti-dsDNA autoantibody in the plasms (**Figure 6C**). Reduction of urinary protein was found at day 15 and day 30 from the start of treatment (**Figure 6D**). Kidneys from the mice treated with GSK126 exhibited smaller glomeruli with reduced hypercellularity, less mesangial expansion, and weak staining for the deposition of immune complex (**Figure 6E**, left panel). The histopathological scores in GSK126 group were lower than those in vehicle group (**Figure 6E**, right panel). As expected, the expression of two representative ISGs (ISG15 and IFIT3) was decreased in GSK126 group (**Figure 6F**). We did not observe any statistically significant changes of spleen size (**Figure S4A**) and alteration of T cell subsets by GSK126 (**Figures S4B-D**). Thus, GSK126 improved the survival and mitigated lupus nephritis while reducing the expression of renal ISGs in NZB/NZW F1 mice that were in late stage of disease.

## Discussion

Dysregulation of epigenetic modifications is involved in the pathogenesis of SLE (6–8, 10, 11, 16, 18). Since epigenetic modifications are reversible, epigenetic modification agents may serve as novel drug candidates for SLE. Indeed, epigenetic modification agents have shown promising efficacy in cancers and other autoimmune diseases (40–42). Additionally, the fact that histone modification enzymes can post-translationally modify non-histone protein components of cell signaling pathways (14) indicates epigenetic modification agents have the potential to interfere with multiple pathogenic pathways of SLE.

EZH2 is a histone methyltransferase and mediates the di- and trimethylation of H3K27, which represses gene expression (15). Previous studies have documented that abnormal glycolysis results in excessive expression of EZH2 in CD4+ T cells of SLE patients, which is responsible for the abnormalities of CD4+ T cells (10, 16, 43). While, EZH2 inhibitor reduced T cell number and proinflammatory cytokines and ameliorated lupus-like disease in MRL/lpr mice (18). In addition, EZH2 can also regulate proinflammatory signaling pathways through direct methylation of essential transcription factors (20). Therefore, considering the overactivation of IFN-I signaling pathway is found in more than 60% of SLE patients and critical to the development of SLE (9, 21, 22), we investigated the role of EZH2 on the activation of IFN-I signaling pathway and EZH2 inhibitor’s therapeutic effects in an SLE mouse model which depends on IFN-I.

Our data show that inhibiting EZH2, either by siRNAs or by chemical inhibitors, attenuated the activation of IFN-I signaling pathway, as indicated by reduced induction of ISGs and decreased phosphorylation of STAT1. Microarray analysis suggests EZH2 is involved in the induction of a wide range of ISGs by IFN-I, especially those constitute the 21-gene IFN signature used as a biomarker for evaluating the efficacy of IFN-I blockade (35, 36). These results indicate a previously unexpected non-traditional role of EZH2 on IFN-I signaling pathway, which depends on EZH2’s methyltransferase activity since blocking its methyltransferase activity by GSK126 is sufficient to inhibit the activation of IFN-I signaling.

This seems contrary to EZH2’s role as a gene expression repressor (15). In fact, EZH2 could suppress the expression of ISGs in MCF-7 cells by epigenetically inhibiting IRF9 (44). However, there are also evidence showing EZH2 can act as co-activator of critical transcription factors in nucleus and methylate non-histone proteins in cytosol (45). Of note, a previous study has shown that EZH2 methylates STAT3 and enhances the Y705 phosphorylation and activity of STAT3 in glioblastoma cells (20). Considering STAT1 and STAT3 are both composed of a number of structurally and functionally conserved domains (46), EZH2 may also have the ability to enhance the tyrosine phosphorylation and activity of STAT1, which promotes the activation of IFN-I signaling.

These findings indicate two functionally opposite mechanisms can be used by EZH2 to regulate IFN-I downstream gene expression, which makes it complex to predict the net outcomes of EZH2 inhibition on IFN-I signaling. We propose that, since epigenetic regulation of gene expression is cell type-specific (47), the discrepancy between different studies may be attributed to cell type specificity of EZH2 mediated epigenetic regulation of the genes involved in IFN-I downstream signal transduction or ISGs themselves. Indeed, in the study demonstrating the inhibitory effect of GSK126 on EZH2, the authors have noticed the complexity and uniqueness of EZH2 mediated epigenomic regulation of gene expression in different cell lines (33). They have shown, in the lymphoma cell lines tested, GSK126 reduced the global H3K27me3 levels and activated the transcription of EZH2 target genes with a significant variation observed between the upregulated gene sets. GSK126 induced the expression of 16 ISGs of the 21-gene IFN signature in KARPAS-422 cells, but not in Pfeiffer cells [**Table S8**, data were retrieved from reference (33)], which indicates EZH2 mediated repression of IFN-I downstream gene expression is cell type-specific. Thus, in the cells with less or no repressive modification of the genes of IFN-I signaling pathway or ISGs, EZH2 inhibition may mainly interfere with IFN-I signaling, which overrides its epigenetic effects, leading to a suppressed IFN-I signaling; while, in the cells with heavy repressive modifications, its epigenetic effects may be dominant promoting the expression of ISGs.

Another point is that the study showing EZH2 inhibition promoted the expression of ISGs were performed in tumor cells with no exogenous IFN-I stimulation (44). This is different from the context of SLE, in which IFN-I is overproduced and the cells from immune system or target tissues are constantly stimulated by IFN-I (22). Since in SLE the expression of ISGs are mainly caused by overactivation of IFN-I signaling pathway, EZH2 inhibition will possibly exert an inhibitory effect on ISG expression. On the other hand, for the *in vivo* application of EZH2 inhibitor, the regimen may also contribute to the net outcomes through determining the extent the epigenetic landscape is affected. We have noticed, although only by *in vitro* experiments, long term incubation with EZH2 inhibitor was needed to achieve substantial reduction of H3K27me3 levels and prolonged pretreatment (≥72-h incubation) of cells with EZH2 inhibitor, which mimics high dose and frequent *in vivo* administration of EZH2 inhibitor, indeed increased ISGs expression (data not shown). Therefore, relatively lower dose and less frequent regimen may be better for the treatment of SLE with EZH2 inhibitor, which is different from the treatment of tumors, as it has been shown that less frequent dosing regimen and relatively short half-life of GSK126 limited the effective exposure (48). Interestingly, twice per week intravenous administration of 50 to 3,000 mg of GSK126 did not obviously affect global H3K27me3 ratios in PBMCs as compared with baseline (48). Overall, the above evidence and inference indicate a complex and context dependent mechanism of action for EZH2 inhibition. Not able to depict the exact molecular mechanisms for the regulatory role of EZH2 on IFN-I signaling pathway and for the *in vitro* and *in vivo* effects of EZH2 inhibition is a limit of our study. Future in depth analysis of all possible factors and parameters involved in the context of SLE is warranted, which is necessary to appreciate EZH2’s role in SLE.

The overactivation of IFN-I signaling pathway can be evaluated by measuring the expression of ISGs (21). We found the upregulation of EZH2 associated with the overexpression of ISGs both in PBMCs and in kidney tissues. Thus, we propose that excessive EZH2 contributes to the pathogenesis of SLE at least partly through promoting the activation of IFN-I signaling pathway and EZH2 inhibitor may be used to treat IFN-I-driven SLE manifestations.

We verified the therapeutic effects of EZH2 inhibitor on lupus nephritis in NZB/NZW F1 mice, which depends on IFN-I signaling pathway for disease progression (37–39). Firstly, we proved that the EZH2 inhibitor, GSK126 could inhibit the *ex vivo* and *in vivo* activation of IFN-I signaling pathway. Then, we found GSK126 protected NZB/NZW F1 mice that were in a moderate to severe disease state from death. It reduced the titers of anti-dsDNA autoantibody and 24-h urinary protein and improved renal histopathological changes, while reducing the expression of ISGs in kidneys. Previous reports documented the role of EZH2 on T cell differentiation (18, 49). We did observe a trend of increased Treg cells and decreased DN T cells in the spleen of the NZB/NZW F1 mice treated with the current GSK126 regimen, which was similar to previous studies. But these changes of T cell subsets were not statistically significant. Thus, although it is hard to prove GSK126 solely functions through regulating IFN-I signaling pathway, our data suggest GSK126 exert its therapeutic effects in NZB/NZW F1 mice mainly by interfering with the overactivated IFN-I signaling pathway, while it has a relatively weak effect on T cell centric pathogenic mechanisms.

In summary, our results reveal an unexpected non-traditional function of EZH2 in facilitating the activation of IFN-I signaling pathway, and excessive EZH2 contributes to the overactivation of IFN-I signaling pathway in SLE patients. We prove that EZH2 inhibitor can inhibit the *in vivo* activation of IFN-I signaling pathway and mitigate lupus nephritis in mouse model. Additionally, the heterogeneity of pathogenic mechanisms among SLE patients makes it difficult to develop new therapies (1, 3, 50). While, common regulators of different disease driving pathways can be multivalent therapeutic targets that benefit broad SLE patients (1, 50). Considering EZH2’s regulatory functions in T-cell and B-cell abnormalities of SLE patients (10, 16, 17, 51), we propose that EZH2 is a potent multivalent therapeutic target for SLE and warrants further clinical development.

## Data Availability Statement

The original contributions presented in the study are included in the article/**Supplementary Material**. Further inquiries can be directed to the corresponding authors.

## Ethics Statement

The studies involving human participants were reviewed and approved by the Research Ethics Board of Ren Ji Hospital. The patients/participants provided their written informed consent to participate in this study. The animal study was reviewed and approved by the Animal Care Committee of Ren Ji Hospital.

## Author Contributions

NS, BQ, and LW designed the study, analyzed data, interpreted results, and wrote the manuscript. BQ, LW, and XJ performed experiments. CQ assessed renal pathological scores. CZ collected human samples and clinical data. LW performed bioinformatic data analysis. All authors contributed to the article and approved the submitted version.

## Funding

This work was supported by the National Natural Science Foundation of China (No. 81701603, No. 31630021, No. 31930037), Shanghai Pujiang Program (2019PJD028), Shanghai Municipal Key Medical Center Construction Project (2017ZZ01024-002), National Human Genetic Resources Sharing Service Platform (2005DKA21300), and Innovative research team of high-level local universities in Shanghai, Sanming Project of Medicine in Shenzhen (SZSM201602087).

## Conflict of Interest

The authors declare that the research was conducted in the absence of any commercial or financial relationships that could be construed as a potential conflict of interest.
