# Supplementary material for: EZH2 Inhibition Interferes With the Activation of Type I Interferon Signaling Pathway and Ameliorates Lupus Nephritis in NZB/NZW F1 Mice
Source: Front Immunol. 2021 Mar 26;12:653989. doi: 10.3389/fimmu.2021.653989 (PMC8044841; doi:10.3389/fimmu.2021.653989)
Supplement: Supplementary file 1 [file DataSheet_1.docx]

Supplementary Material

## Supplementary Materials and methods

## RNA-seq analysis

THP-1 cells were transfected with EZH2 targeting siRNAs (si-EZH2) (200 nM) or negative control siRNAs (200 nM). 24 hours later, the cells were stimulated with universal IFN-I (1000U/ml) for 6 hours. Total RNAs were extracted and processed with the RiboMinus™ Human/Mouse Transcriptome Isolation Kit (Invitrogen) to deplete ribosomal RNAs. Then the libraries were prepared with Illumina TruSeq Stranded Total RNA LT Sample Prep Kit. All libraries were size-selected, quantified, and were subjected to deep sequencing with Hiseq 4000 Sequencing Platforms (Illumina). Raw reads were mapped to GRCh37/hg19 human reference genome with HISAT2 (v2.0.4). Transcript expression levels were calculated by kallisto (v0.43.0) with reference gene annotations (Gencode version 19). Differential gene expression analysis was performed in R (v3.3.2) with DESeq2 package (v1.14.1).

## Flow cytometry analysis of T cell subsets

NZB/NZW F1 mice were sacrificed 2 days after the last GSK126 treatment (Figure 5A) and the spleens were harvested. Spleen single-cell suspensions were obtained by grinding the spleen followed by filtration through a cell strainer (pore size 70 μm) (Corning). The cells were treated with red blood cell lysis buffer (Beyotime Biotechnology) and washed with PBS. Then, they were resuspended in staining buffer at a concentration of 1 x 106 per 100μl and stained with the fluorochrome-conjugated antibodies for 30 min at 4 ℃. Antibodies used were purchased from BD Biosciences or Biolegend and listed in Table S5. Cells were analyzed with a BD LSRFortessaTM Flow Cytometer (BD Biosciences). The gating strategy is shown in Figure S4B. We defined the CD3+CD4+CD8- population as CD4+ T cells, the CD3+CD4-CD8+ population as CD8+ T cells, CD3+CD4-CD8- population as double negative T cells, the CD3+CD4+CXCR3+CCR6- population as Th1 cells, the CD3+CD4+CXCR3-CCR6+ population as Th17 cells, the CD3+CD4+CXCR3+CCR6+ population as pathogenic Th17 cells, the CD3+CD4+CD25+CD127- population as Treg cells.

## Supplementary Figures


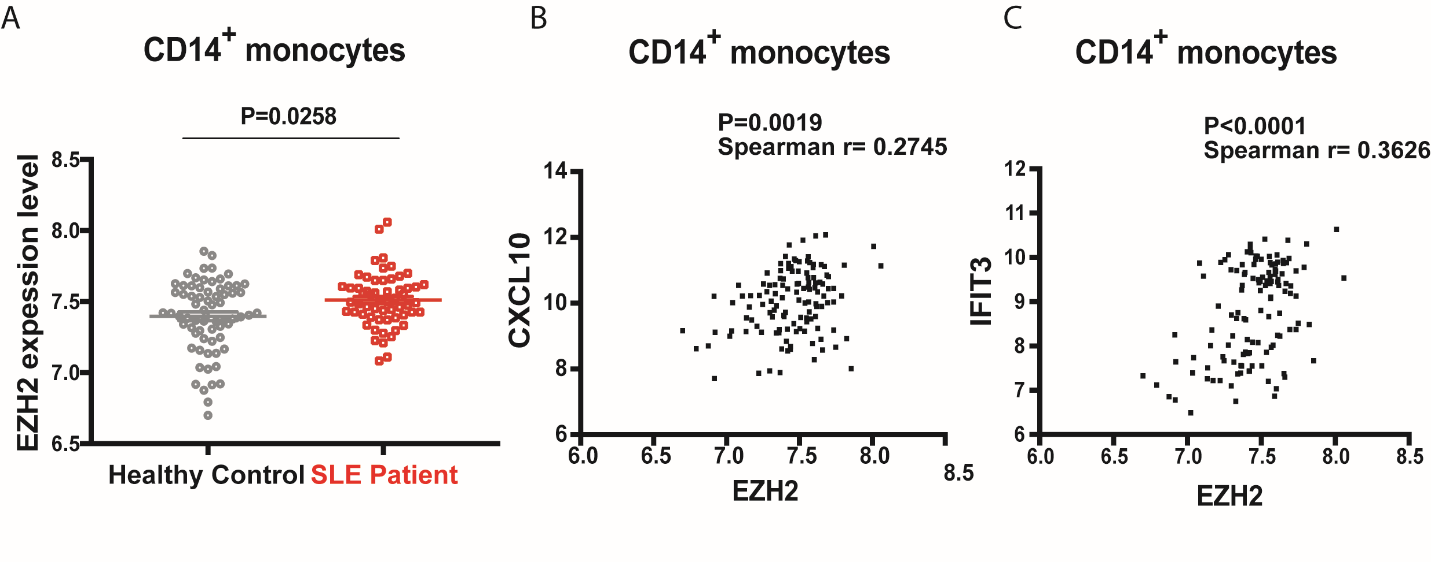
**Figure S1. Overexpression of EZH2 associates with the upregulation of ISGs in primary monocytes from SLE patients.** (A) The expression levels of EZH2 in primary CD14^+^ monocytes from SLE patients (n = 58) and healthy controls (n = 68). (B and C) Positive correlation between the expression levels of EZH2 and CXCL10 (B) and IFIT3 (C) in primary CD14^+^ monocytes from all subjects including SLE patients and healthy controls (n = 126). The expression levels of EZH2, CXCL10 and IFIT3 were generated from the microarray dataset (ArrayExpress, accession number E-MTAB-2713). (A, B and C) Each dot represents individual subjects. (A) Data are presented as mean ± SEM. *P* values were determined by Mann-Whitney U-test (A) or by Spearman's correlation test (B and C).


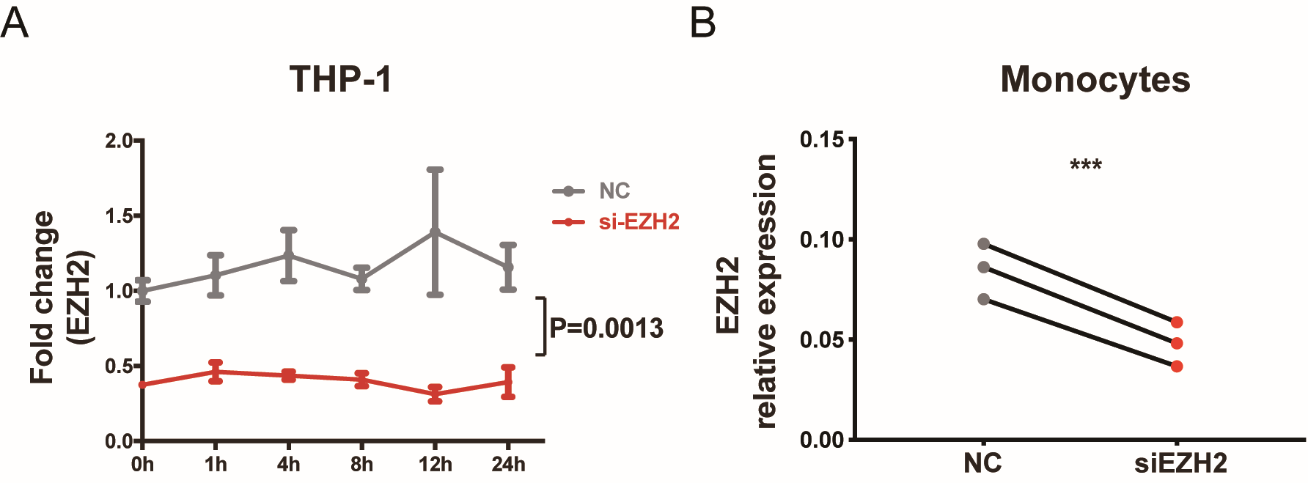


**Figure S2. The efficacy of EZH2 targeting siRNAs.** The levels of EZH2 mRNA in THP-1 cells (A) or primary monocytes (B) transfected with 200nM of EZH2 targeting siRNAs (siEZH2) or negative control siRNAs (NC).


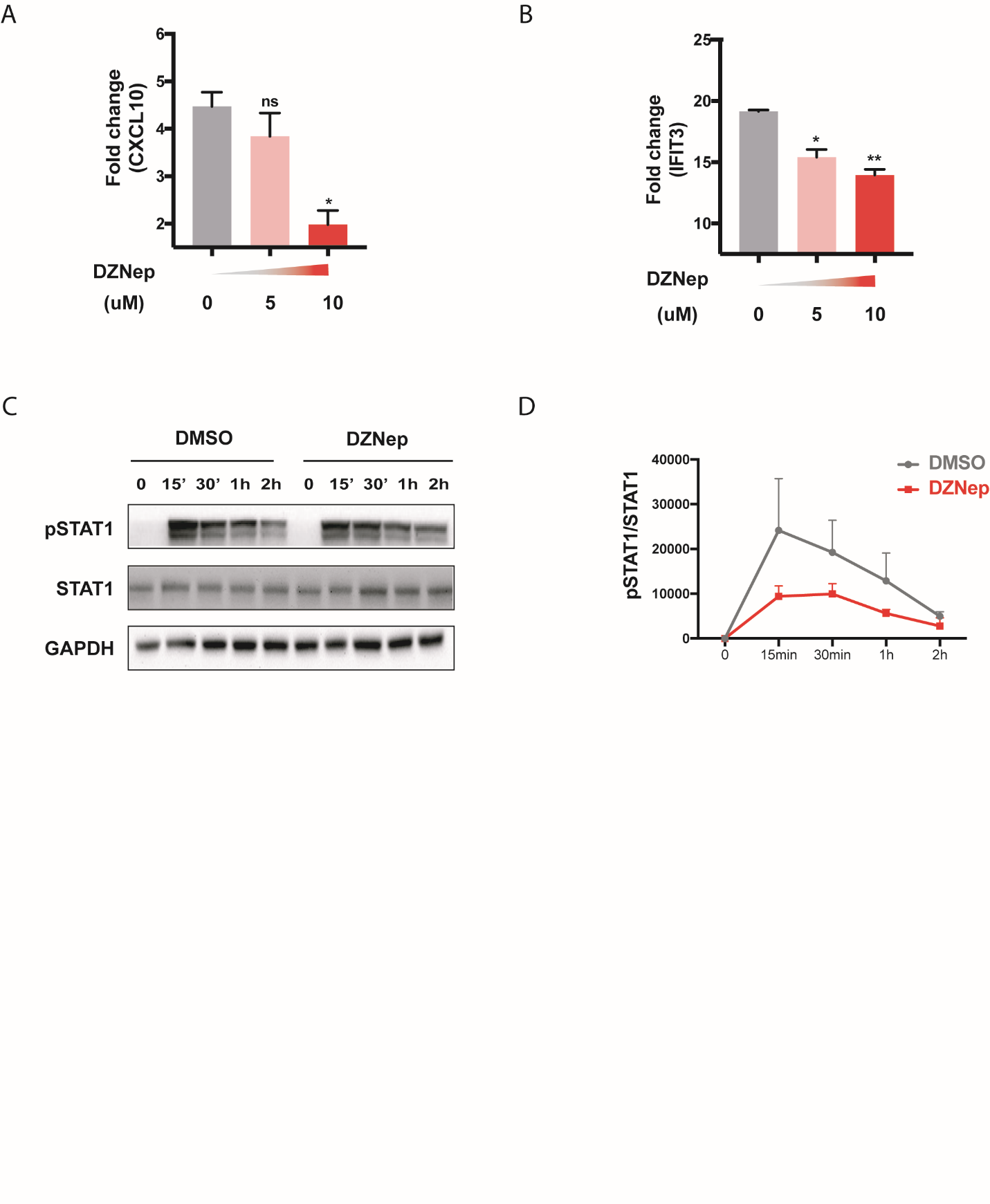


**Figure S3. DZNep attenuates the activation of IFN-I signaling pathway.** (A and B) THP-1 cells were pretreated with different concentration of DZNep for 30 min, then stimulated with universal IFN-I (1000U/ml) for 4 hours. The levels of CXCL10 mRNA (A) and IFIT3 mRNA (B) were measured. (C and D) IFN-I induced phosphorylation of STAT1 was determined in THP-1 cells pretreated with 10 μM of DZNep by Western bolt. (C) Representative pictures of at least 3 independent experiments. (D) Band densities quantified by Image J. (A, B and D) Data are presented as mean ± SEM. *P* values were determined by Mann-Whitney U-test. * *P* < 0.05, ** *P* < 0.01, ns: not significant.


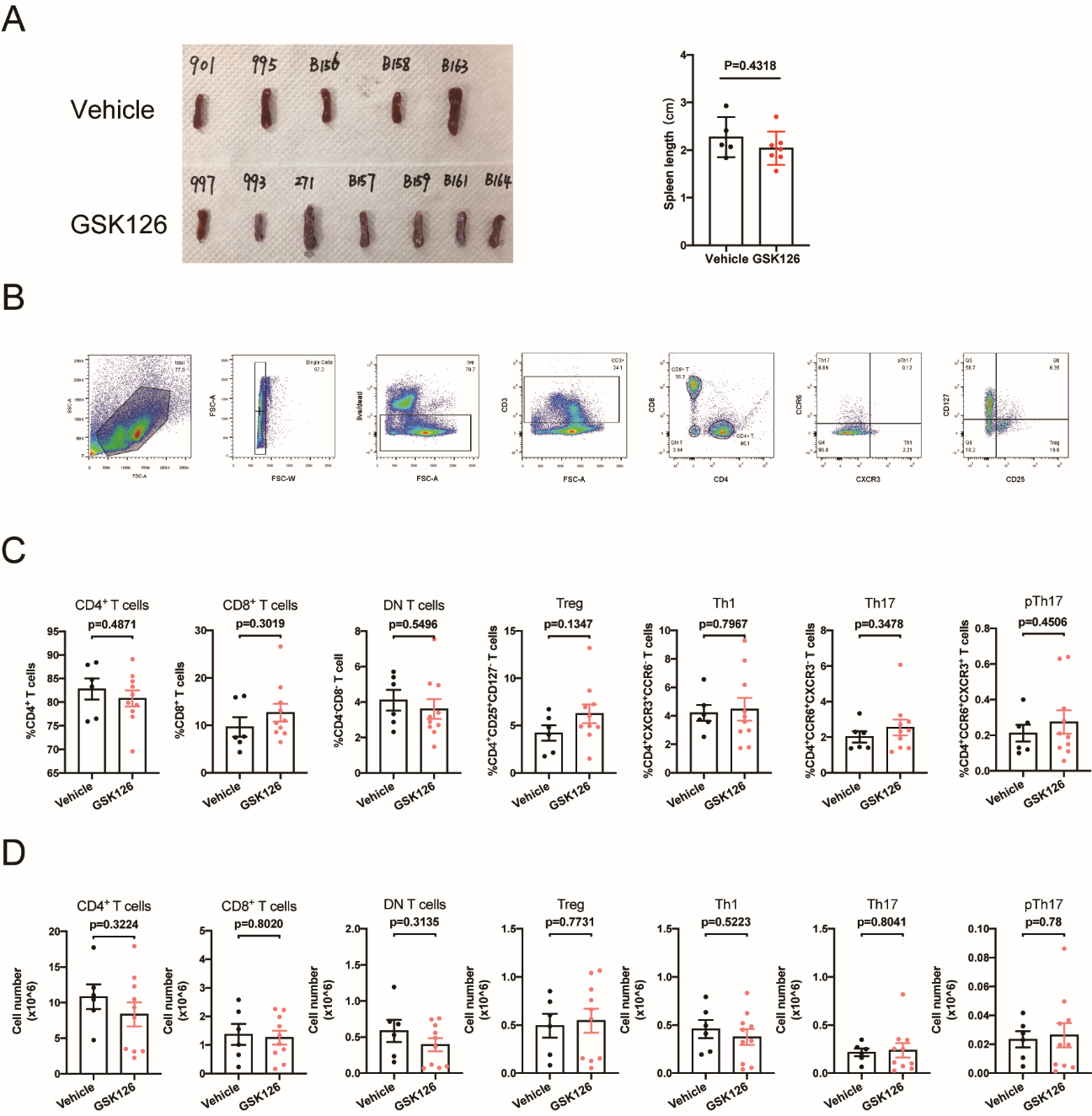


**Figure S4. Flow cytometry analysis of the T cell subsets in the spleen of NZB/NZW F1 mice.** (A) Representative photo of the spleens from the NZB/NZW F1 mice in GSK126 group and vehicle control group. Spleen length were measured. (B) Gating strategy for T cell subsets in spleen. (C) Percentages of different T cell subsets in the spleen from the NZB/NZW F1 mice in GSK126 group and vehicle control group. (D) Absolute cell numbers of different T cell subsets in the spleen from the NZB/NZW F1 mice in GSK126 group and vehicle control group. (A, C and D) Each dot represents individual mice. Data are presented as mean ± SEM. *P* values were determined by Mann-Whitney U-test.

## Supplementary Tables

**Table S1. Clinical information of the study subjects.**

|  | Healthy controls  (n=30) | SLE patients  (n=30) |
| --- | --- | --- |
| Sex, No. male/No. female | 3/27 | 3/27 |
| Age, mean ± SD | 36 ± 9 | 34 ± 10 |
| Disease duration, mean ± SD (months) |  | 84.23 ± 75.03 |
| SLEDAI score, mean ± SD |  | 8 ± 4 |

**Table S2. Sequences of siRNAs used in this study.**

| siRNA | Sequence (5’-3’ sense/ 3’-5’ antisense) |
| --- | --- |
| EZH2 siRNA-1 sense | CAAAGAAUCUAGCAUCAUATT |
| EZH2 siRNA-1 antisense | TTGUUUCUUAGAUCGUAGUAU |
| EZH2 siRNA-2 sense | GAGGACGGCUUCCCAAUAATT |
| EZH2 siRNA-2 antisense | TTCUCCUGCCGAAGGGUUAUU |
| EZH2 siRNA-3 sense | GCUGAAGCCUCAAUGUUUATT |
| EZH2 siRNA-3 antisense | TTCGACUUCGGAGUUACAAAU |
| EZH2 siRNA-4 sense | GAAUGGAAACAGCGAAGGATT |
| EZH2 siRNA-4 antisense | TTCUUACCUUUGUCGCUUCCU |
| NC sense | UUCUCCGAACGUGUCACGUTT |
| NC antisense | TTAAGAGGCUUGCACAGUGCA |

**Table S3. Sequences of qPCR primers used in this study.**

| Genes | Sequence (5’-3’) |
| --- | --- |
| hsa-RPL13a forward primer | GGGCAGGTTCTGGTATTGGAT |
| hsa-RPL13a reverse primer | GGCTCGGAAGTGGTAGGGG |
| hsa-EZH2 forward primer | TGATGGAGACGATCCTGAAG |
| has-EZH2 reverse primer | TTCTGCTGCCCTTATCTCG |
| hsa-ISG15 forward primer | ACTCATCTTTGCCAGTACAGGAG |
| hsa-ISG15 reverse primer | CAGCATCTTCACCGTCAGGTC |
| hsa-CXCL10 forward primer | TTCTGATTTGCTGCCTTATC |
| hsa-CXCL10 reverse primer | CTTGGATTAACAGGTTGATTACT |
| hsa-IFIT3 forward primer | TGAGGTCACCAAGAATTCCCTG |
| hsa-IFIT3 reverse primer | CAATCTGGTTACACACTCTATCTTC |
| mus-RPL13a forward primer | GGGCAGGTTCTGGTATTGGAT |
| mus-RPL13a reverse primer | GGCTCGGAAGTGGTAGGGG |
| mus-ISG15 forward primer | AGAGCAAGCAGCCAGAAG |
| mus-ISG15 reverse primer | CACCGTCATGGAGTTAGTCAC |
| mus-IFIT3 forward primer | TAAGGAAGTCAGAGCACAATG |
| mus-IFIT3 reverse primer | GTCTCACCTTGTCAACGTAA |

**Table S4. Antibodies used in western blot.**

| Antibody | Company | Cat. |
| --- | --- | --- |
| HRP-conjugated GAPDH | Abclonal | AC035 |
| Anti-STAT1 (phospho Y701) | Abcam | ab29045 |
| STAT1antibody | Cell Signaling Technology | 9172 |

**Table S5. FACS antibodies.**

| Antibody | Company | Cat. |
| --- | --- | --- |
| Fixable viability stain 780 | BD Bioscience | 565388 |
| BV510 hamster anti-mouse CD3e | BD Bioscience | 563024 |
| PerCP anti-mouse CD4 Antibody | Biolegend | 100538 |
| PE-Cy7 Rat Anti-mouse CD8a | BD Bioscience | 552877 |
| Brilliant Violet 605 anti-mouse CD183a (CXCR3) antibody | biolegend | 1265523 |
| PE anti-mouse CD196 (CCR6) antibody | biolegend | 129804 |
| FITC rat anti-mouse CD25 | BD Bioscience | 553072 |
| Brilliant Violet 421 anti-mouse CD127 (IL-7Ra) antibody | biolegend | 135023 |

**Table S6. Reduction fold for the genes of the top 20 affected ISGs in Figure 2C.**

| Genes | Reduction fold (siEZH2(+)/siNC(+)) |
| --- | --- |
| RIN2 | 0.30 |
| CASK | 0.56 |
| DPYS | 0.39 |
| MEP1B | 0.30 |
| SMTNL1 | 0.43 |
| ENPP2 | 0.48 |
| SIDT1 | 0.31 |
| CACNA1I | 0.35 |
| SLC30A4 | 0.49 |
| CCL8 | 0.14 |
| GBP1 | 0.14 |
| CXCL10 | 0.09 |
| NTRK2 | 0.57 |
| CXCL11 | 0.11 |
| HAPLN3 | 0.29 |
| PLEKHA4 | 0.33 |
| SECTM1 | 0.42 |
| IDO1 | 0.17 |
| ISG20 | 0.42 |
| CSF1 | 0.32 |
| TNFSF10 | 0.45 |

**Table S7. Reduction fold for the genes of the 21-gene IFN-I signature in Figure 2C.**

| Genes | Reduction fold (siEZH2(+)/siNC(+)) |
| --- | --- |
| RSAD2 | 0.44 |
| USP18 | 0.51 |
| ISG15 | 0.61 |
| IFIT3 | 0.72 |
| IFIT1 | 0.65 |
| MX1 | 0.68 |
| PLSCR1 | 0.70 |
| EPSTI1 | 0.72 |
| OAS1 | 0.70 |
| LY6E | 0.72 |
| IFI27 | 0.69 |
| HERC5 | 0.76 |
| RTP4 | 0.74 |
| IFI6 | 0.77 |
| OAS2 | 0.88 |
| IFI44L | 0.93 |
| IFI44L | 0.94 |
| OAS3 | 0.96 |
| SPATS2L | 1.02 |
| SIGLEC15 | 1.02 |
| LAMP3 | 1.41 |

**Table S8. Induction fold for the genes of 21-gene IFN-I signature by GSK126 in two different lymphoma cell lines.**

| Induction fold by GSK126 in lymphoma cell lines^#^ | | |
| --- | --- | --- |
| Genes | KARPAS-422 | Pfeiffer |
| RSAD2 | 44.4 | NS^*^ |
| USP18 | 28.4 | NS |
| ISG15 | NS | NS |
| IFIT3 | 18.5 | NS |
| IFIT1 | 17.9 | NS |
| MX1 | 2.4 | NS |
| PLSCR1 | 3.5 | NS |
| EPSTI1 | 6.5 | 3 |
| OAS1 | 4.4 | NS |
| LY6E | NS | 2 |
| IFI27 | 31.9 | NS |
| HERC5 | NS | NS |
| RTP4 | 107.5 | NS |
| IFI6 | 5.6 | NS |
| OAS2 | 15.5 | NS |
| IFI44L | 69.2 | NS |
| OAS3 | 4.5 | NS |
| SPATS2L | 34.6 | 2.2 |
| SIGLEC15 | NS | NS |
| LAMP3 | 2.9 | NS |

^#^Data were retrieved from supplementary table 6 of reference (35).

^*^NS: No fold change data was found for the gene, because it was filtered out by the cut-off criteria based on *P* value and average fold change. Therefore, NS indicates no significant change for the gene.
